# Comparing multidisciplinary and brief intervention in employees with different job relations on sick leave due to low back pain: protocol of a randomised controlled trial

**DOI:** 10.1186/s12889-017-4975-3

**Published:** 2017-12-16

**Authors:** Pernille Pedersen, Claus Vinther Nielsen, Morten Hovgaard Andersen, Vivian Langagergaard, Anders Boes, Ole Kudsk Jensen, Chris Jensen, Merete Labriola

**Affiliations:** 1 Section of Clinical Social Medicine and Rehabilitation, Department of Public Health, Aarhus University, P.P. Oerums Gade 11, 1B, Silkeborg, 8600 Aarhus, Denmark; 2 DEFACTUM, Central Denmark Region, Aarhus, Denmark; 3 Spine Center, Diagnostic Center, Silkeborg Regional Hospital, Silkeborg, Denmark; 4 Department of Clinical Social Medicine & Rehabilitation, The Regional Hospital West Jutland, Aarhus, Denmark; 5 Department of Public Health and General Practice, Norwegian University of Science and Technology, NTNU, Trondheim, Norway; 6 National Advisory Unit on Occupational Rehabilitation, Rauland, Norway

**Keywords:** Return to work, Sick leave, Low back pain, Multidisciplinary intervention, Brief intervention, Job relations

## Abstract

**Background:**

Low back pain (LBP) is a common problem that affects the lives of many individuals and is a frequent cause of sickness absence. To help this group of individuals resume work, several interventions have been studied. However, not all individuals may profit from the same intervention and the effect of a given intervention on return to work (RTW) may depend on their work situation. The aim of this study is to evaluate whether employees on sick leave due to LBP and with poor job relations will benefit more from a multidisciplinary intervention, while patients with strong job relations will benefit more from a brief intervention.

**Methods:**

The study is designed as a randomised controlled trial with up to five years of follow-up comparing brief intervention with brief intervention plus multidisciplinary intervention. Employees, aged 18–60 years, are included in the study from March 2011 to August 2016 if they have been on sick leave for 4–12 weeks due to LBP with or without radiculopathy. They are divided into two groups, a group with poor job relations and a group with strong job relations based on their answers in the baseline questionnaire. Each group is randomised 1:1 to receive the brief intervention or brief intervention plus multidisciplinary intervention. The brief intervention comprises a clinical examination and advice offered by a rheumatologist and a physiotherapist, whereas the supplementary multidisciplinary intervention comprises the assignment of a case manager who draws up a rehabilitation plan in collaboration with the participant and the multidisciplinary team.

The primary outcome is duration of sickness absence measured by register data. Secondary outcomes include sustainable RTW and questionnaire-based measures of functional capacity. Outcomes will be assessed at one, two and five years of follow-up.

**Discussion:**

This trial will evaluate the effect of brief and multidisciplinary intervention on RTW and functional capacity among employees on sick leave due to LBP with poor or strong job relations. This will indicate whether work-related characteristics should be considered when providing treatment of LBP patients in the health care sector.

**Trial registration:**

Current Controlled Trials ISRCTN14136384. Registered 4 August 2015.

## Background

Low back pain (LBP) is a common health problem that most individuals experience at some point in their life. Worldwide, it causes disability more frequently than any other condition. For the individual, it can cause limitations in everyday activity and an increase in absence from work. Thereby, it imposes economic burdens not only for the individuals and their families, but also for society as a whole [1]. More than half of the individuals with LBP will recover within a year, but many of the individuals with LBP and limitations in activity will experience recurrent episodes that may be longer in duration and associated with greater disability [2]. Therefore, sickness absence is frequently caused by LBP [3]. The reduced workability is not solely attributed to pain, but is also influenced by psychological factors, health concerns, and coping strategies [4]. As the underlying factors are multidimensional, all individuals on sick leave due to LBP may not profit from the same intervention to improve their workability. A brief intervention including a clinical examination and reassuring advice given by a rheumatologist and a physiotherapist has proven effective in facilitating return to work (RTW) in some studies [5–7]. Other studies have shown positive effects on RTW associated with more comprehensive multidisciplinary interventions including, e.g., work place visits, cognitive therapy, and physical rehabilitation programs [8–12]. Recently we compared the effect of a multidisciplinary and a brief intervention in employees on sick leave due to LBP, where no difference in RTW rates was found between the two interventions after one, two and five years of follow-up [13–15]. However, in a subgroup analysis differences in RTW rates were found in employees with different work situations [16]. Employees feeling at risk of losing their job due to their current sick leave or with no influence on planning their work (poor job relations) had a higher RTW rate when offered the multidisciplinary intervention. Inversely, employees with some job control and no fear of losing their job (strong job relations) returned to work quicker when offered the brief intervention. Employees, who blamed their work for causing LBP and claimed economic compensation in addition to regular sick-leave benefits, did not return to work faster when receiving the multidisciplinary intervention irrespective of the job relation. Poor work outcomes for this group have also been reported in other studies [12, 17].

However, post-hoc subgroup analyses are problematic and should be verified in randomised trials. We decided to initiate a new randomised intervention study to test the findings from the previous subgroup analyses.

### Aim

The aim of this study is to evaluate, whether employees on sick leave due to LBP and with poor job relations, will benefit more from a multidisciplinary intervention, while employees with strong job relations will benefit more from a brief intervention.

### Hypothesis

For employees who have poor job relations i.e. feeling at risk of losing their job and/or with no perceived influence on job planning and who have not claimed additional economic compensation for their disease or injury, it is hypothesized that the multidisciplinary intervention compared to the brief intervention will:

- support the employees to a faster return to work
- support a sustainable RTW
- improve the employees’ functional capacity

For employees who have strong job relations i.e. not feeling at risk of losing their job and with influence on job planning or who have claimed economic compensation for their disease or injury, it is hypothesized that the brief intervention compared to the multidisciplinary intervention will:

- support the employees to a faster return to work
- support a sustainable RTW
- improve the employees’ functional capacity

## Methods/Design

### Study design, procedure, and participants

The study is designed as a randomised controlled trial (RCT) comparing brief intervention with brief intervention plus multidisciplinary intervention with up to five years of follow-up for two groups of employees (a group with poor job relations and a group with strong job relations). The interventions take place at the Research Unit of the Spine Center, Regional Hospital Silkeborg, Denmark, from March 2011 to August 2016. General practitioners in 13 municipalities with a total of 750.000 citizens receive written information about the study and are encouraged to refer employees who are aged 18–60 years, have been partly or fully on sick leave from work for 4 to 12 weeks because of LBP with or without radiculopathy, and are able to read and speak Danish. The first visit takes place at the Spine Center within two weeks after the referral. The employees are not enrolled in the study if they have continuing or progressive signs of radiculopathy implicating plans for surgery, had low back surgery within the last year or specific back diseases, are pregnant, have known dependency on drugs or alcohol or have any primary psychiatric disease.

### Randomization and blinding

**Fig. 1:**
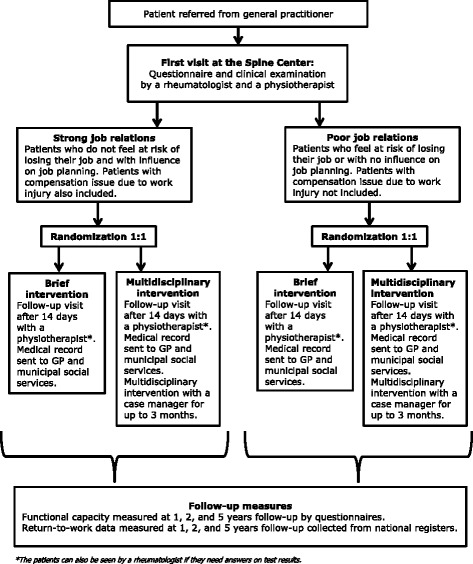
Design of the study

At the first visit at the Spine Center the participants complete a baseline questionnaire before the initial clinical examination. Based on the answers of the questionnaire the participants are divided into the group of poor job relations or the group of strong job relations **(**Fig. 1 **)**. Each group is randomised 1:1 to receive brief intervention or brief intervention plus multidisciplinary intervention. A secretary randomises the participants based on a computerized random number generator. The initial clinical examination is double-blinded, but afterwards both participants and caregivers are aware of the results of the randomization. The analyses of the study will be performed blinded.

### Interventions

The rationale and goal behind this intervention is that some or all participants who are at risk of significant limitations in their physical, mental and / or social functioning due to low back pain, can achieve an independent and meaningful life.

The approach of the intervention providers is inspired by different domains of knowledge, primarily the bio-psycho-social back pain model by Waddell 1998 [18], but also the knowledge on low back pain treatment and the approach presented by Indahl 2004 [19], Loisel et al. 1997 [20] and Anema et al. 2007 [11]. Also elements from psychology is incorporated, among others cognitive-behavioural therapy [21] and communication skills [22].

The multidisciplinary intervention uses the ICF-model as a basic instrument by the multidisciplinary team [23], which contributes to a better understanding of the participants’ functioning on the basis of a bio-psycho-social model. Also a systematic approach inspired by The Extended Reattribution and Management Model (TERM model) is used in order to optimize communication skills. This model is built on a consultation process and is based on these four steps a) Understanding, b) The physician’s expertise and acknowledgement of illness, c) Negotiating the participants’ understanding of low back pain d) Negotiating further treatment [22].

To ensure a standardized intervention, supervision of the team is performed by a general practitioner specialized in cognitive therapy.

The procedures; all participants will receive a clinical examination by a rheumatologist and a physiotherapist meeting them with curiosity and recognition. MRI of the lumbar spine will be available at the first visit, but the imaging will not be seen by the rheumatologist or explained to the participant until after the clinical examination.

The rheumatologist records a medical history and performs a standard clinical low back examination including measurement of range of motion and estimation of diffuse tenderness by tender point examination. Furthermore, the participant will be examined for signs indicating radiculopathy. If symptoms and signs are in accordance with MRI-findings, the participant will be classified as having radiculopathy. Other relevant imaging and examinations will be available if considered necessary. The often-poor consistency between imaging and back pain in general will be explained to the participants. The participants will be informed about the beneficial effect of all kinds of exercises being the best-documented treatment for LBP [24]. In addition, the participant will be informed that psychosocial stress, worrying - and possibly depression - can cause worsening and prolongation of pain. If depression is present and prominent, anti-depressant therapy will be offered in cooperation with the general practitioner. After the clinical examination, the imaging is demonstrated to the participant. Degenerative manifestations will be explained as possible contributors to pain, except when they are in accordance with the nerve root affected in which cases the pain could be explained. Reassuring explanations for back and leg pain and advice to gradually increase physical activity will be given to the participants. If there is no dangerous finding in the spine, the participants will get the following information: “You may do as much as you can”. If radiculopathy is present, the participant will be told, that it is safe to be physically active, as long as the exercises / activity do not worsen leg pain. Furthermore, the participant will be informed about the good prognosis for disc herniation in general and about the possibility of surgery if no improvement occurs. Finally, the participant will also be advised about red flags indicating urgent evaluation from a physician and eventually referral to a back surgeon. Medical pain management will be adjusted if necessary and the participants are advised to resume work when possible.

Afterwards, the medical history and the results of the clinical examination are communicated to the physiotherapist in the presence of the participant. The physiotherapy examination includes a standardized, mechanical evaluation (MDT), a standardized stretching evaluation, a standardized dynamic and static exercise evaluation, and advice on exercise. The participant is asked to estimate the percentage of usual activity he/she is able to do. All participants are advised to start exercising aerobically and gradually increasing dosage, and the physiotherapist helps the participant to choose type of exercise. They will be encouraged to increase physical activity to 3–4 h per week. A participant with directional preference causing centralization of the pain is advised accordingly. The participant will be recommended to complete an exercise diary for use at the follow-up visit 2 weeks later.

A copy of the medical record is sent to the participants, the general practitioner, and the municipal social services responsible for reimbursement of sick leave benefits. For all participants a follow-up visit with the physiotherapist will be arranged two weeks later, and a follow-up visit with the rheumatologist will be arranged for participants who need feedback on tests. For participants allocated to brief intervention the intervention is closed at this point and the general practitioner takes over if necessary.

For participants allocated to multidisciplinary intervention, a visit with a case manager is scheduled within a week after the first consultation. The case manager conducts a comprehensive interview covering aspects of work history, present work tasks, physical and psychological working environment, private life and influence of pain and disability on work functioning. Furthermore, the interview focuses on psychological pain management and any uncertainties regarding RTW. If relevant, the participant is referred to the staff psychologist concerning appropriate pain management or other personal issues. The participant and the case manager make a tailored rehabilitation plan to facilitate RTW. The plan includes realistic goals regarding workload, specific working terms and time for, e.g., partial resumption of work.

The case manager contacts the municipal job centre to discuss and coordinate the plan. Also, the workplace will be contacted if the participant agrees to the arrangement of a meeting at the workplace. The aim is to negotiate a specific time schedule for gradual RTW and a realistic workload arranged in accordance with the employer. The entire multidisciplinary team will discuss the rehabilitation plan in an attempt to address all relevant bio-psycho-social considerations concerning RTW. The team includes a specialist of social medicine, a rheumatologist, a social worker, a physiotherapist, an occupational therapist and in relevant cases a psychologist. If necessary, the participants can be scheduled for a meeting with one or more of these professionals. The case manager keeps in contact with the participant and each case will be discussed at team conferences depending on need and progress. When the participant has resumed work, or if this, after three months, seems impossible, the case is closed. The case manager can either be the specialist of social medicine, the social worker, or the occupational therapist.

### Questionnaires

At baseline the participants are asked about their job relations based on two questions;

"Do you have influence on work planning?" and "Do you feel at risk of losing your job due to the present sick leave?". Participants who answer “yes” to the first question and “no” to the second, are in the “strong job relation” group, while participants who answer either “no/yes”, “yes/yes” or “no/no” are in the “poor job relation” group. Furthermore, the participants are asked "Have you claimed for compensation because of your work-related illness or health?". Those who answer “yes” are in the “strong job relation” group.

At baseline, before the initial clinical examination, and at one, two and five years of follow-up the participants complete a questionnaire. The questions are related to health, functional capacity, social aspects, workplace factors, and individual factors. The following standardized instruments are used to assess health and functional capacity: The Low Back Pain Rating scale [25], the Orebro Musculoskeletal Pain Questionnaire [26], the Roland Morris disability scale [27], the Euroqol (EQ5-d) [28], the Short Form-12 (SF-12) [29], the Common Mental Disorder Questionnaire (CMDQ) [30], and the Major Depression Inventory (MDI) [31].

### Primary outcome

Time to RTW is the primary outcome of the study, and is defined as the period between randomisation and to RTW for at least 4 consecutive weeks without sickness absence recurrence. RTW is operationalized as not receiving any social transfer income except unemployment benefits, and will be measured by data from The Danish National Labour Market Authority’s DREAM database [32], which provides weekly information on all public transfer payments.

### Secondary outcomes

The total number of weeks on sick leave will be measured during one, two and five years of follow-up. The same will the “Work participation score” (WPS), defined as a fraction with numbers of weeks working as the numerator and numbers of weeks receiving social transfer payments + numbers of weeks working as the denominator [33].

Furthermore, the percentages of participants with RTW recorded after one, two, and five years will also be calculated.

These outcomes will be measured with data from the DREAM database.

Pain intensity is measured by the Low Back Pain Rating scale [25], which includes three questions about pain in the lower back and three similar questions about pain in the legs. The three questions include actual pain, worst pain and average pain during the last two weeks. The pain is measured on an 11-point rating scale ranging from “no pain” (0) to “worst possible pain” (10). The sum of the answers is used for scoring (0–60).

Fear-avoidance belief is measured by three questions from the Orebro Musculoskeletal Pain Questionnaire [26]. The questions examine to what degree physical activity is believed to increase the pain, and rated from 0 (completely disagree) to 10 (completely agree), thus the sum score can range from 0 to 30.

The Roland Morris disability scale [27] is used to measure the present functional level. It includes 23 questions about the ability to perform daily activities, and is measured as “no” (0) or “yes” (1), with a sum score from 0 to 23.

Health-related quality of life is measured by Euroqol (EQ5-d) [28], which consists of five questions related to current mobility, self-care, activity, social relationship, pain and mood. It is scored on a three-point rating scale.

Self-rated health is measured by the Short Form-12 (SF-12) version 1 [29], which consists of 12 questions that correspond to eight subscales and two major summary scores, a physical component summary (PCS) and a mental component summary (MCS). The score of each major domain will be used and ranges from 0 to 100; the higher the scores, the higher the levels of functioning.

Mental health related to concern, anxiety, depression, and somatoform disorders is measured by the Common Mental Disorder Questionnaire (CMDQ) [30]. It consists of 32 items scored on a five-point Likert scales (0 = not at all, 4 = extremely) to detect the severity of psychiatric symptoms within the last four weeks. Questions about alcohol abuse are omitted in this study.

The Major Depression Inventory (MDI) [31] is used to assess depression. It consists of 10 items with a six-point Likert scale to estimate the level of depressive symptoms from “at no time” (0) to “all the time” (5), with a sum score from 0 to 50.

### Power calculation

Duration of sickness absence until full RTW was chosen as the primary outcome measure and was used for sample size calculation. Data from subgroup analyses of the previous study at Silkeborg Spine Centre [13, 14, 16] showed that participants with strong job relations had a higher change of RTW when receiving the brief intervention with a hazard ratio of 0.66. In contrast, data showed that participants with poor job relations had a higher chance of RTW when receiving the multidisciplinary intervention and power calculations were based on a hazard ratio of 1.5.

A sample size calculation based on the log rank-method showed that 99 employees with strong job relations and 102 employees with poor job relations were needed in each intervention group, thus a total of 198 and 204 employees, respectively, were needed for inclusion.

The calculation was based on a one-sided significance level of 5% and a power of 80%.

### Analysis

The following analyses will be performed for each group of participants; i.e. comparing effects between interventions for those with poor and for those with strong job relations.

The rates of RTW will be compared between the two interventions at one, two and five years after randomization by means of Cox regression. The duration of sick leave will be calculated by counting the total number of weeks with sick leave during each year of follow-up. Differences between groups will be tested with the Wilcoxon rank-sum test. The WPS will be accumulated over the years and the median score will be compared between groups by means of Wilcoxon rank-sum test. Moreover, differences between interventions in frequency of participants with a WPS above 75% will be calculated using Chi^2^-test as well as the relative risk of having a score less than 75%. The percentages of participants with RTW recorded at year one, two, and five will be calculated and tested between groups by means of logistic regression.

Moreover, any other effects on health and functional capacity between interventions will be measured at the same time points and assessed by appropriate tests according to the distribution of data. Comparisons of baseline characteristics of responders and non-responders to the follow-up questionnaires will be made.

The analyses will be performed using STATA 13 IC (Stata Corp, College Station, TX), and a significance level of *p* < 0.05 will be considered statistically significant.

All analyses will primarily be performed on an intention-to-treat basis; however, per-protocol analyses will also be performed.

### Ethical considerations

Participation is voluntary, and project information is given both verbally and in writing. The participants are informed about their rights to decline participation and to withdraw with no consequences in terms of their sickness absence benefits. Moreover, they are informed about the consequences of randomization and group allocation.

All participants sign informed consent in relation to their participation in the study and to the use of their health data. The participants are offered to bring a companion throughout the course of the study.

Previous research has indicated that neither of the two types of interventions induces risk to the participants. Furthermore, results have shown that none of the interventions are preferable for all participants in relation to reduce sickness absence. The results of the study will be published regardless of the outcome.

All participants are assigned an identification number and will be treated anonymously in all analyses. Papers and electronic documentation with names and personal identification numbers are stored securely in locked cabinets or on a password-protected computer.

## Discussion

This trial will evaluate the effect of a brief and a multidisciplinary intervention on RTW and functional capacity among employees on sick leave due to LBP with poor or strong job relations. The trial focuses on offering different treatment to different employees by dividing them into two groups at the outpatient hospital clinic according to their job relations. Based on the results of this trial we will gain more insight into how to treat employees on sick leave to facilitate RTW and restore functional capacity.

The major strength of this study is the randomised design and the large group of participants. The clinical examinations by the rheumatologist and the physiotherapist are performed double blinded, which ensures impartiality and reduces treatment bias. Register data will be used to measure RTW, which is preferable compared to self-reported data in regard to receiving more accurate information on the sick leave period and avoid missing data [34]. Validated instruments are used to cover both physical and mental aspects of health and functional capacity.

The first results will be available for analysis in 2017.
